# Effect of istradefylline on postural abnormalities in patients with Parkinson's disease: An association study of baseline postural angle measurements with changes in Unified Dystonia Rating Scale total score

**DOI:** 10.1016/j.ensci.2023.100493

**Published:** 2023-12-30

**Authors:** Makio Takahashi, Toshio Shimokawa, Jinsoo Koh, Takao Takeshima, Hirofumi Yamashita, Yoshinori Kajimoto, Hidefumi Ito

**Affiliations:** a Department of Neurology, Kitano Hospital, The Tazuke-Kofukai Medical Research Institute, Osaka, Japan; b Clinical Study Support Center, Wakayama Medical University, Wakayama, Japan; c Department of Neurology, Wakayama Medical University, Wakayama, Japan; d Department of Neurology, Tominaga Hospital, Osaka, Japan; e Department of Neurology, Japanese Red Cross Wakayama Medical Center, Wakayama, Japan; f Department of Neurology, Wakayama Medical University Kihoku Hospital, Wakayama, Japan

**Keywords:** Angle value, Istradefylline, Parkinson's disease, Postural abnormalities, Unified Dystonia Rating Scale

## Abstract

In our previous study, istradefylline treatment in patients with Parkinson's disease (PD) improved postural abnormalities (PAs), as seen from a decrease in the mean Unified Dystonia Rating Scale (UDRS) total score from week 0 to week 24. A subgroup analysis based on baseline clinical characteristics investigated the association between improvement in the UDRS total score and istradefylline treatment. However, the association between an objective assessment of PAs and improvement in the UDRS total score is unclear. This ad hoc analysis investigated the association between improvement in the UDRS total score after istradefylline treatment and baseline trunk and neck angles, objective assessments of PAs, measured from patients' photographs taken in the previous study. The patients (*n* = 31) were stratified into groups based on the trunk forward flexion angle (TFFA), trunk lateral flexion angle (TLFA), and neck flexion angle (NFA) values at baseline. From week 0 to week 24, significant improvements in the UDRS total score were found in median percent change (−8.33% [interquartile range: −43.97, 0.00], *P* = 0.039) in patients with equal to or above the median TFFA values, and in median change (−‍1.50 [−9.25, 0.00], *P* = 0.015) and median percent change (−13.33% [−50.47, 0.00], *P* = 0.009) in patients with equal to or above the median TLFA values. Patients with more advanced PAs showed more consistent improvements in the UDRS total score with istradefylline. Baseline TFFA and TLFA values, which are objective values, may be useful to assess the istradefylline effectiveness in patients with PD and PAs.

## Highlights

• Analysis of istradefylline in Parkinson's disease with postural abnormalities (PAs).
• Earlier, istradefylline improved Unified Dystonia Rating Scale (UDRS) total score.
• Association between baseline trunk and neck angles and changes in UDRS total score.
• Subgroups based on below and equal to or above median trunk and neck angle values.
• More advanced PAs showed more consistent improvements with istradefylline in UDRS.

## 1 Introduction

Parkinson's disease (PD) is often accompanied by postural abnormalities (PAs), such as anterior and lateral flexion of the thoracolumbar spine and antecollis, in approximately 20% of patients [1,2]. Compared with European patients with PD, Asian patients tend to develop PAs, particularly antecollis, earlier in the disease course [3]. Additionally, camptocormia is a critical disability in patients with PD, commonly observed in the advanced stage [4]. As PAs become irreversible after the development of secondary changes, such as muscle atrophy and bone fracture, recognizing and appropriately managing PAs in their early stages is necessary for better prognosis [5], but there is insufficient information to do so.

Levodopa is widely used in the treatment of patients with PD, and some reports have demonstrated the efficacy of levodopa for PAs in these patients, but its effectiveness is limited and still controversial [5,6]. In our previous study, we investigated the efficacy and safety of istradefylline, a nondopaminergic, selective adenosine A_2A_ receptor antagonist, for the treatment of PAs in patients with PD who were experiencing the wearing-off phenomenon on levodopa-containing therapies [7]. The mean (95% confidence interval [CI]) Unified Dystonia Rating Scale (UDRS) total score, which is a tool to assess dystonia and was designed to include 14 body regions for rating, allowing more detailed evaluations of dystonia, improved significantly from 15.2 (12.4, 18.0) at baseline (week 0) to 10.2 (7.7, 12.8) at week 24 (mean change: 4.84 [95% CI: 1.97, 7.71], *P* = 0.001), with significant improvements in the neck, right distal arm and hand (including elbow), and trunk severity scores at week 24 compared with baseline, suggesting that istradefylline could be efficacious for treating PAs in patients with PD [7]. Furthermore, a subgroup analysis based on the baseline clinical characteristics of patients was used to investigate the association between an improvement in the UDRS total score and istradefylline treatment, which revealed that female sex, age < 75 years, Mini-Mental State Examination (MMSE) score ≥ 27 points, and modified Hoehn and Yahr (mH&Y) score ≥ 2 were associated with an improvement in the UDRS total score after istradefylline treatment [7]. Istradefylline significantly improved the UDRS total score both in patients with and without therapy with dopamine agonists [7]. However, the subgroup analysis of the previous study did not include an objective assessment of the symptoms of PAs, and further investigation using an objective assessment method was warranted. In clinical practice, various methods are employed to evaluate PAs in patients with PD, such as clinical scale, wall goniometer, and photo-based measurements [8].

In this study, we aimed to investigate the association between the improvement in the UDRS total score and baseline angles of the trunk and neck by using an objective assessment estimated from photographs of patients with PD and PAs treated with istradefylline in the previous study. To determine the efficacy of istradefylline for different severities of PAs, we divided the patients into two subgroups based on below and equal to or above the median value of trunk and neck angles at baseline to compare the differences in improvement in the UDRS total score for each group.

## 2 Methods

### 2.1 Study design

This was an ad hoc analysis of a multicenter, open-label, single-arm, 24-week prospective and exploratory study that enrolled adult patients with PD and PAs experiencing the wearing-off phenomenon associated with levodopa-containing therapies (study period: September 2016–November 2019) [7]. In the previous study, patients were initiated on oral istradefylline 20 mg once daily (QD), with a subsequent dose increase to 40 mg QD at week 4, if tolerated well. If tolerability was impaired owing to adverse drug reactions, the dose of istradefylline was reduced to 20 mg QD after week 4 [7]. During the study, changes to the dosage and administration schedule as well as addition of new therapeutic agents for the treatment of PD were not permitted; however, dosage reduction of istradefylline and existing concomitant medications was permitted if there were any tolerability issues due to adverse drug reactions [7]. Interventions that the investigators considered to have an impact on the evaluation of PAs (e.g., enteral solution containing levodopa/carbidopa hydrate; neurosurgery, such as deep brain stimulation, transcranial magnetic stimulation, electroconvulsive therapy, and stereotactic neurosurgery; treatment with botulinum toxin and other drugs affecting the assessment of PAs; and other novel physical therapies, such as exercise) were prohibited during the study. This study was conducted in accordance with the Declaration of Helsinki (Revised, Fortaleza, October 2013; translated by the Japanese Medical Association) and the Ethical Guidelines for Biological Sciences and Medical Research for People (enforced in 2021), as well as other laws and regulations related to this study. The study protocol was approved by the Medical Ethics Committee of Kitano Hospital (P210701200) and registered in the University Hospital Medical Information Network (UMIN) Clinical Trials Registry (UMIN000045364). All enrolled patients provided written informed consent. However, if patients had difficulty in writing due to their condition, representatives were allowed to provide written consent on their behalf, subject to their prior oral consent [7].

### 2.2 Eligibility criteria

Patients whose photographs had been taken in the previous study, who had provided written informed consent, and whose numerical angle data had been acquired were eligible for inclusion in this ad hoc study.

### 2.3 Outcomes

The outcomes of interest were the change and percent change in the UDRS total score from week 0 to week 24 of istradefylline treatment.

### 2.4 Assessments

To objectively assess the severity of PAs before istradefylline treatment (baseline; week 0), the values of the trunk forward flexion angle (TFFA), trunk lateral flexion angle (TLFA), and neck flexion angle (NFA) were measured from photographs of the patient's standing position at baseline and at week 24 of istradefylline treatment.

By fixing the camera position at the same level as that of the patient's waist and by fixing the shooting conditions such as magnification, all photographs were captured under the same conditions every time. In addition, multiple trained measurers identified the positions of C7 and L5 and double-checked the angle measurement to ensure data accuracy. All the photographs were captured when the patient's symptoms were in the “ON” state. Based on the angle values at baseline, the patients were stratified into two subgroups for each body area: those with angle values below the median and those with angle values equal to or above the median. Improvement in PAs was assessed using the median change and median percent change in the UDRS total score from week 0 to week 24 in each subgroup according to the severity of PAs at baseline.

### 2.5 Methods of measurements

**Fig. 1:**
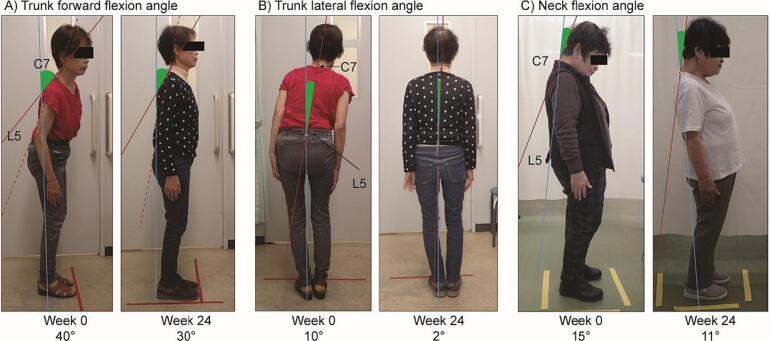
Method of measurement of A) trunk forward flexion angle (TFFA), B) trunk lateral flexion angle (TLFA), and C) neck flexion angle (NFA): Representative photographs of patients at baseline and at 24 weeks of istradefylline treatment. The green shaded area shows the angle of measurement.

The TFFA, TLFA, and NFA values before istradefylline treatment were measured from the right and left lateral views and from the dorsal view (Fig. 1). For TFFA and NFA, the averages of the flexion angles from both sides were used in the analysis. For NFA, positive angle values indicated forward flexion, and negative angle values indicated dorsiflexion.

#### 2.5.1 Measurement of TFFA

The TFFA value was measured using the photographs of both the lateral sides [9].

① A straight line was drawn through the cervical spine (C7) and lumbar spine (L5; dotted red line).
② A straight line through the point at the back most distal to the straight line in step ① and the cervical spine (C7) was used as the oblique axis (solid red line). When it was difficult to draw the oblique axis in this manner, a straight line through the greater trochanter and acromion was used as the oblique axis.
③ A perpendicular line drawn from “the point at the back most distal to the straight line in step ①” as determined in step ② to the floor was used as the base axis (blue line). When drawing a line perpendicular to the floor, structures such as columns, window frames, and floor lines in the photographs were used as reference points.
④ The angle between the base and oblique axes was measured as the TFFA value (green shaded area).

#### 2.5.2 Measurement of TLFA

The TLFA value was measured using the photographs from the dorsal side.

① A line perpendicular from the lumbar spine (L5) to the floor was used as the base axis (blue line). When drawing a line perpendicular to the floor, structures such as columns, window frames, and floor lines in the photograph were used as reference points.
② A straight line through the cervical spine (C7) and lumbar spine (L5) was used as the oblique axis (red line). When it was difficult to draw the oblique axis in this manner, a straight perpendicular line passing through the acromion on both sides was used as the oblique axis.
③ The angle between the base and oblique axes was measured as the TLFA value (green shaded area).

#### 2.5.3 Measurement of NFA

The NFA value was measured using the photographs of both the lateral sides.

① A straight line through the cervical spine (C7) and lumbar spine (L5) was used as the base axis (blue line).
② A straight line through the cervical spine (C7) and occipital region of the head was used as the oblique axis (red line). When it was difficult to determine the oblique axis in this manner, a straight line through the external ear foramen and parietal region of the head was used as the oblique axis.
③ The angle between the base and oblique axes was measured as the NFA value (green shaded area).

### 2.6 Statistical analysis

The analysis set was the efficacy analysis set used in the previous study [7]; however, patients for whom photographs could not be acquired were excluded from the study. Normal distribution was examined using a histogram and a normal probability plot. When an assumption of normal distribution was not feasible, statistical analysis was evaluated using the Wilcoxon signed rank sum test, and the median values of variation and rate of change and interquartile range (IQR) were calculated. The UDRS total score was assessed as the median change and median percent change from week 0 to week 24. A subgroup analysis was performed using demographics and baseline clinical characteristics as quantitative variables. Missing data were handled as is, and no specific imputation was performed. However, if missing data for individual patients were determined in the previous study, these data were followed in the same manner. For patients with outlier values that could not be considered medically, if the previous study determined a way of handling them, these values were followed in the same manner. Statistical analysis was conducted using R version 4.2.1 (The R Foundation for Statistical Computing, Vienna, Austria), and two-sided *p*-values <0.05 were considered statistically significant.

## 3 Results

### 3.1 Baseline angle values before istradefylline treatment

Up to 31 patients whose photographs had been taken in the previous study were included in this analysis. Before istradefylline treatment, the median (IQR) TFFA (*n* = 26), TLFA (*n* = 31), and NFA (n = 26) values were 37.00° (33.50, 42.25), 3.00° (1.00, 5.50), and −21.00° (−28.88, −13.12), respectively.

### 3.2 Demographics and baseline clinical characteristics of patients before istradefylline treatment

The median TFFA, TLFA, and NFA values were calculated from the angle values at baseline (Fig. 1), and the patients were categorized into two subgroups: 1) below the median and 2) equal to or above the median. Patient characteristics before istradefylline treatment were comparable in both the subgroups for all the three angles (Supplementary Table 1).

### 3.3 Median change and median percent change from baseline in the median UDRS total score in patients by postural angles

At week 0, the median (IQR) UDRS total scores in patients with equal to or above the median values vs those with below the median values were 11.00 (9.00, 18.00) vs 18.00 (11.00, 19.00) for TFFA, 18.00 (10.00, 19.00) vs 12.00 (9.25, 18.00) for TLFA, and 18.00 (9.00, 19.00) vs 13.00 (10.00, 19.00) for NFA (Table 1). The median UDRS total scores at week 24 decreased compared with those before treatment for TFFA, TLFA, and NFA.

**Table 1:** UDRS total score before and after istradefylline treatment stratified by postural angles.

	UDRS total score			Change in UDRS total score	*p*-value⁎	Percent change in UDRS total score	*p*-value⁎
	Week 0		Week 24				
Trunk forward flexion angle (Median: 37°)	Equal to or above the median value	11.00 (9.00, 18.00)	10.00 (7.25, 12.50)	−0.50 (−6.25, 0.00)	0.062	−8.33 (−43.97, 0.00)	**0.039**
	Below the median value	18.00 (11.00, 19.00)	11.00 (7.00, 18.00)	−2.00 (−11.00, 0.00)	0.109	−10.00 (−53.85, 0.00)	0.218
Trunk lateral flexion angle (Median: 3°)	Equal to or above the median value	18.00 (10.00, 19.00)	12.00 (6.00, 18.00)	−1.50 (−9.25, 0.00)	**0.015**	−13.33 (−50.47, 0.00)	**0.009**
	Below the median value	12.00 (9.25, 18.00)	8.00 (7.50, 10.50)	−2.00 (−7.50, 0.00)	0.054	−20.00 (−57.48, 0.00)	0.109
Neck flexion angle (Median: −21°)	Equal to or above the median value	18.00 (9.00, 19.00)	11.00 (9.00, 18.00)	−1.00 (−6.00, 1.00)	0.103	−10.00 (−35.29, 5.56)	0.147
	Below the median value	13.00 (10.00, 19.00)	7.00 (2.25, 12.25)	−3.50 (−7.25, 0.00)	0.125	−26.92 (−77.50, 0.00)	0.125

Data are presented as median (IQR).

IQR, interquartile range; UDRS, Unified Dystonia Rating Scale.

⁎ Wilcoxon signed rank sum test (week 24 vs week 0). *p*-values that were significant at a significance level of α = 0.05 are shown in bold font.

A significant difference was observed in the median (IQR) percent change in the UDRS total score (−8.33% [−43.97, 0.00], *P* = 0.039) from week 0 to week 24 in patients with equal to or above the median values for TFFA. In patients with equal to or above the median values for TLFA, the median (IQR) change in the UDRS total score (−1.50 [−9.25, 0.00], *P* = 0.015) and its median percent change (−13.33% [−50.47, 0.00], *P* = 0.009) also showed a statistically significant difference from week 0 to week 24 (Table 1).

## 4 Discussion

In this ad hoc analysis, patients were subgrouped according to the severity of PAs based on the angle values of the trunk and neck (below the median and equal to or above the median) before istradefylline treatment, and the median change and median percent change in the UDRS total score from week 0 to week 24 of each subgroup were also determined. The median percent change in the UDRS total score from week 0 to week 24 significantly improved in patients with median or above values for TFFA and TLFA at baseline. These results suggest that patients with more advanced symptoms of PAs at baseline showed more consistent improvements in the UDRS total scores after the 24-week istradefylline treatment. Although the change in the UDRS total score in patients with below-median values for TFFA and TLFA did not reach statistical significance, a remarkable decrease was noted in the UDRS total score at week 24 compared with that at week 0, suggesting that PAs may be improved with istradefylline treatment even in patients with mild symptoms. The reason why there was no statistically significant difference in changes in the UDRS total score in patients with below the median values for TFFA and TLFA may be because the UDRS total score did not consistently improve within the group. On the other hand, the statistically significant difference observed in the patients with median and above values for TFFA and TLFA may be attributed to the consistent improvement in the UDRS total score within these groups.

This ad hoc analysis investigated the effect of istradefylline on the improvement in the UDRS total score in patients with PD and different severities of PAs prior to treatment. Clinical impressions before the start of treatment allow physicians to anticipate improvements in the patient's symptoms. The strength of this study is that it established the utility of the UDRS total score for the assessment of PAs in patients with PD. Although the UDRS total score is a subjective method of assessment of PAs evaluated by clinicians, it can be reliably used in clinical practice as it is sensitive to changes with treatment by objective photograph-based judgment. However, this study also has a few limitations. First, the patients' static posture, but not dynamic posture was evaluated in this study. As camptocormia and other PAs tend to worsen with movement, the dynamic posture measurement could have triggered more prominent camptocormia. Therefore, the evaluation of PAs by video monitoring and captured images could have overcome this limitation. Second, careful interpretation of the study results is warranted considering the relatively small sample size.

## 5 Conclusion

This ad hoc analysis investigated the association of the improvement with istradefylline in the UDRS total score with baseline angles of the trunk and neck. Significant changes in the UDRS total score were observed in patients with more severe PAs at baseline. The results suggest that patients with more advanced PA symptoms showed more consistent improvements in the UDRS total scores with istradefylline. This study provides a useful reference for istradefylline prescribing in real-world clinical practice.

## Funding

This work was supported by Kyowa Kirin Co., Ltd.

## Author contributions

M.T. contributed substantially to the conception, organization, and execution of the trial; review and critique of the statistical analysis; writing of the first draft; and review and critique of the manuscript. T.S. was involved in the conception of the trial; design, execution, review, and critique of the statistical analysis; and review and critique of the manuscript. J.K., T.T., H.Y., and Y.K. were involved in the execution of the trial and review and critique of the statistical analysis and the manuscript. H.I. was involved in the conception, organization, and execution of the trial and review and critique of the statistical analysis and the manuscript.

## CRediT authorship contribution statement

**Makio Takahashi:** Writing – review & editing, Writing – original draft, Visualization, Supervision, Resources, Project administration, Methodology, Investigation, Funding acquisition, Formal analysis, Conceptualization. **Toshio Shimokawa:** Writing – review & editing, Writing – original draft, Visualization, Validation, Software, Resources, Methodology, Investigation, Formal analysis, Data curation, Conceptualization. **Jinsoo Koh:** Writing – review & editing, Resources, Investigation. **Takao Takeshima:** Writing – review & editing, Resources, Investigation. **Hirofumi Yamashita:** Writing – review & editing, Resources, Investigation. **Yoshinori Kajimoto:** Writing – review & editing, Resources, Investigation. **Hidefumi Ito:** Writing – review & editing, Resources, Investigation, Formal analysis, Conceptualization.

## Declaration of competing interest

None.

## Appendix A Supplementary data

Supplementary material: Demographics and baseline clinical characteristics stratified by postural angle before istradefylline treatment. Supplementary material
