# Supplementary material for: Effect of istradefylline on postural abnormalities in patients with Parkinson's disease: An association study of baseline postural angle measurements with changes in Unified Dystonia Rating Scale total score
Source: eNeurologicalSci. 2023 Dec 30;34:100493. doi: 10.1016/j.ensci.2023.100493 (PMC10809095; doi:10.1016/j.ensci.2023.100493)
Supplement: Supplementary file 1 — Supplementary material: Demographics and baseline clinical characteristics stratified by postural angle before istradefylline treatment. [file mmc1.docx]

**Supplementary Table 1** Demographics and baseline clinical characteristics stratified by postural angle before istradefylline treatment.

|  | TFFA (n = 26) | | TLFA (n = 31) | | NFA (n = 26) | |
| --- | --- | --- | --- | --- | --- | --- |
|  | Equal to or above the median value | Below the median value | Equal to or above the median value | Below the median value | Equal to or above the median value | Below the median value |
| Age, years | 74.26  (71.46, 79.15) | 77.24  (73.15, 79.24) | 73.40  (69.78, 78.39) | 76.86  (71.78, 79.52) | 78.39  (72.77, 79.24) | 73.41  (66.32, 77.24) |
| Sex, n (%) |  |  |  |  |  |  |
| Male | 6 (46.15) | 6 (46.15) | 5 (29.41) | 8 (57.14) | 8 (61.54) | 4 (30.77) |
| Female | 7 (53.84) | 7 (53.84) | 12 (70.59) | 6 (42.86) | 5 (38.46) | 9 (69.23) |
| Height, cm | 153.60 (149.00, 160.40) | 155.30 (149.00, 162.00) | 151.00 (148.40, 160.00) | 153.20 (149.78, 160.00) | 160.00  (152.50, 163.10) | 151.00  (147.90, 154.30) |
| Weight, kg | 47.30  (43.00, 59.90) | 56.70  (46.00, 58.90) | 46.00  (39.80, 58.90) | 47.80  (47.08, 56.77) | 57.90  (47.30, 60.00) | 47.00  (39.00, 55.20) |
| BMI, kg/m^2^ | 20.13  (18.48, 21.46) | 21.77  (20.18, 22.62) | 20.95  (17.98, 22.62) | 20.39  (19.10, 22.06) | 21.46  (19.50, 22.62) | 20.18  (17.57, 21.73) |
| Duration of disease, years | 5.00  (1.00, 10.00) | 7.00  (2.00, 21.00) | 5.00  (1.00, 13.00) | 5.00  (1.00, 21.00) | 5.00  (3.00, 8.00) | 5.00  (1.00, 21.00) |
| Duration of motor complications, years | 5.00  (1.00, 10.00) | 5.50  (2.00, 21.00) | 5.00  (3.00, 13.00) | 7.00  (1.00, 21.00) | 5.00  (3.00, 8.00) | 5.00  (1.00, 21.00) |
| mH&Y score (on state) | 4.00  (1.00, 5.00) | 4.00  (1.00, 5.00) | 4.00  (1.00, 5.00) | 3.50  (1.00, 5.00) | 4.00  (1.00, 5.00) | 5.00  (1.00, 5.00) |
| mH&Y score (off state) | 4.00  (3.00, 6.00) | 5.00  (2.00, 5.00) | 5.00  (3.00, 6.00) | 3.00  (2.00, 4.00) | 4.00  (2.00, 5.00) | 4.50  (3.00, 6.00) |
| Caregiver presence, n (%) |  |  |  |  |  |  |
| Yes | 10 (76.92) | 7 (53.85) | 11 (64.71) | 10 (71.43) | 9 (69.23) | 8 (61.54) |
| No | 3 (23.08) | 6 (46.15) | 6 (35.29) | 4 (28.57) | 4 (30.77) | 5 (38.46) |
| MMSE score | 29.00  (28.00, 30.00) | 26.00  (25.00, 28.00) | 28.00  (26.00, 29.00) | 28.50  (25.00, 29.00) | 28.00  (26.00, 29.00) | 28.00  (25.00, 29.00) |

Data are presented as median (IQR) unless otherwise specified.

BMI, body mass index; IQR, interquartile range; mH&Y, modified Hoehn and Yahr; MMSE, Mini-Mental State Examination; NFA, neck flexion angle; TFFA, trunk forward flexion angle; TLFA, trunk lateral flexion angle.
